# Factors associated with outcomes of second-line treatment for *EGFR*-mutant non-small-cell lung cancer patients after progression on first- or second-generation EGFR-tyrosine kinase inhibitor treatment

**DOI:** 10.3389/fonc.2023.1104098

**Published:** 2023-06-20

**Authors:** Cheng-Yu Chang, Chung-Yu Chen, Shih-Chieh Chang, Ching-Yi Chen, Yi-Chun Lai, Chun-Fu Chang, Yu-Feng Wei

**Affiliations:** ^1^ Division of Chest Medicine, Department of Internal Medicine, Far Eastern Memorial Hospital, New Taipei City, Taiwan; ^2^ Nursing Department, Cardinal Tien Junior College of Healthcare and Management, New Taipei City, Taiwan; ^3^ Department of Internal Medicine, National Taiwan University Hospital Yunlin Branch, Douliou, Taiwan; ^4^ College of Medicine, National Taiwan University, Taipei, Taiwan; ^5^ Division of Chest Medicine, Department of Internal Medicine, National Yang-Ming Chiao Tung University Hospital, Yi-Lan, Taiwan; ^6^ Faculty of Medicine, College of Medicine, National Yang-Ming Chiao Tung University, Taipei, Taiwan; ^7^ Department of Critical Care Medicine, National Yang-Ming Chiao Tung University Hospital, Yi-Lan, Taiwan; ^8^ Division of Chest Medicine, Department of Internal Medicine, E-Da Hospital, Kaohsiung, Taiwan; ^9^ School of Medicine for International Students, College of Medicine, I-Shou University, Kaohsiung, Taiwan; ^10^ Department of Internal Medicine, E-Da Cancer Hospital, I-Shou University, Kaohsiung, Taiwan

**Keywords:** epidermal growth factor receptor-tyrosine kinase inhibitor, neutrophil-to-lymphocyte ratio, non-small-cell lung cancer, osimertinib, re-biopsy, second-line

## Abstract

**Purpose:**

Epidermal growth factor receptor-tyrosine kinase inhibitors (EGFR-TKIs) are standard first-line treatments for advanced *EGFR*-mutant non-small-cell lung cancer (NSCLC) patients. However, factors associated with outcomes after progression on first-line therapy are seldom investigated.

**Materials and methods:**

From January 2016 to December 2020, we enrolled 242 EGFR-mutant stage IIIB–IV NSCLC patients who progressed on first- or second-generation EGFR-TKI treatments, and 206 of them receive second-line treatments after disease progression. The factors that predict the survival outcomes of different second-line treatments after disease progression were evaluated. Clinical and demographic characteristics, including metastatic sites, neutrophil-to-lymphocyte ratio (NLR) at first-line progression, and second-line treatment regimens, and whether re-biopsied after disease progression or not, were reviewed for outcome analysis.

**Results:**

The univariate analysis showed that the PFS was shorted in male patients (p =0.049), patients with ECOG performance state ≥ 2 (p =0.014), former smokers (p =0.003), patients with brain metastasis (p =0.04), second-line chemotherapy or EGFR-TKIs other than osimertinib (p =0.002), and NLR ≥5.0 (p=0.024). In addition, second-line osimertinib was associated with longer OS compared to chemotherapy and other EGFR-TKI treatment (p =0.001). In the multivariate analysis, only second-line osimertinib was an independent predictor of PFS (p =0.023). Re-biopsy after first-line treatment was associated with a trend of better OS. Patients with NLR ≥5.0 at disease progression had shorter OS than patients with NLR <5.0 (p = 0.008).

**Conclusion:**

The benefits of osimertinib necessitate that aggressive re-biopsy after progression on first- or second-generation EGFR-TKI treatment is merited for appropriate second-line treatments to provide better outcomes for these patients.

## Highlights

- Epidermal growth factor receptor-tyrosine kinase inhibitors (EGFR-TKIs) are standard first-line treatments for advanced *EGFR*-mutant non-small-cell lung cancer (NSCLC) patients. Factors associated with the outcomes in NSCLC patients with disease progression on first- or second-generation EGFR-TKI like gefitinib, erlotinib, or afatinib, have rarely been investigated.
- We enrolled 242 patients treated with gefitinib, erlotinib, or afatinib as first-line treatment. Upon disease progression, only 70 (28.9%) patients underwent re-biopsy and 206 (85.1%) received second-line treatment. Outcome analysis indicated a better outcome in patients who underwent re-biopsy or received osimertinib as the second-line treatment or whose neutrophil-to-lymphocyte ratio was < 5 at disease progression on first-line treatment.
- Aggressive re-biopsy after progression on gefitinib, erlotinib, or afatinib treatment is merited for appropriate second-line treatment such as osimertinib to provide better outcomes for patients.

## Introduction

Lung cancer is the leading cause of mortality due to cancer in the world, with an estimated 1.8 million deaths in 2020.(1) Non-small-cell lung cancer (NSCLC) accounts for 80–90% of all lung cancer cases, and more than 70% of NSCLC patients present with locally advanced or metastatic disease (Stage III or IV) at initial diagnosis.(2) Epidermal growth factor receptor (*EGFR)* mutations are observed in 40–60% and 10–20% of NSCLC patients in Asian and non-Asian populations, respectively.(3) For patients with advanced *EGFR*-mutant NSCLC, EGFR-tyrosine kinase inhibitors (TKIs) are the standard first-line treatment. TKIs have been reported to show a higher response rate and longer progression-free survival (PFS) compared to conventional chemotherapy.(4–6) Osimertinib, a third-generation ECGR-TKI, is the preferred treatment recommended in NCCN guidelines.(7) However, in the FLAURA study, osimertinib did not demonstrate an overall survival (OS) benefit in Asian population subgroups and patients with the *L858R* mutation compared to first-generation EGFR-TKI.(8, 9) In addition, osimertinib may not be available or affordable in some countries due to its relatively high cost.

In patients treated with first- or second-generation EGFR-TKIs, such as gefitinib, erlotinib, and afatinib, disease progression inevitably occurred after a median time of 10 to 14 months. Of these patients, approximately 50% developed EGFR T790M as the resistance mechanism, which could be effectively treated with osimertinib as a subsequent-line treatment.(10) Nevertheless, the detection of EGFR T790M or other resistance mechanisms for the next line of treatment relies on re-biopsy of the tissue or liquid biopsy. The ESMO guidelines recommend switching to platinum-based doublet chemotherapy for patients who cannot undergo tissue biopsy or for those in whom the T790M mutation is not detected.(11)

Previous studies reported that approximately 70% of patients received subsequent treatment after progressing on first- or second-generation EGFR-TKI treatment. Although the re-biopsy rate (tissue or liquid) was 85–87% after disease progression, only 30–46% of patients tested positive for the T790M mutation and received osimertinib treatment.(12, 13) A retrospective, real-world study from Greece showed a T790M positivity rate of 21.9% (based on cobas^®^ molecular testing of plasma and/or tissue biopsy), which may compromise the clinical outcome due to the lack of subsequent osimertinib therapy.(14) However, in patients with the T790M mutation, no statistically significant OS benefit was observed for osimertinib compared to platinum–pemetrexed chemotherapy.(10) Data on factors related to the safety and efficacy of different second-line treatments and their impacts on the prognosis of *EGFR*-mutant NSCLC are limited. The aim of this study was to investigate the factors associated with the efficacy and prognosis in *EGFR*-mutant NSCLC patients who received second-line treatment after progression on first- or second-generation EGFR-TKIs.

## Materials and methods

### Patient selection and data collection

This was a multicenter, retrospective study that included a medical center and three regional hospitals in Taiwan. Between January 2016 and December 2020, patients fulfilled the following criteria were enrolled in the study: **1)** a diagnosis of locally advanced or metastatic (Stage IIIb–IV) *EGFR*-mutant NSCLC; **2)** first- or second-generation EGFR-TKI including gefitinib, erlotinib, or afatinib administered as the first-line treatment; and **3)** confirmed disease progression on first-line EGFR-TKI treatment. Patients who switched to another anti-cancer drug or regimen due to intolerance or reasons other than disease progression were excluded. This study was approved by the Institutional Review Boards of all participating institutions.

Demographic and clinical data related to lung cancer were collected, including age, sex, smoking status, cancer staging at diagnosis, initial metastatic sites, *EGFR* mutation subtype, the type of EGFR-TKI therapy, Eastern Cooperative Oncology Group- Performance Status (ECOG PS) score, neutrophil-to-lymphocyte ratio (NLR) at disease progression on first-line treatment, whether or not re-biopsied after disease progression on first-line and subsequent treatment regimens (including osimertinib, other EGFR-TKIs, and different chemotherapeutic drugs). Other EGFR-TKIs include those patients with treatment beyond progression or switched to TKIs other than osimertinib, or those who have declined or are contraindicated to chemotherapy. NLR was obtained after disease progression on the first-line treatment, which was calculated by dividing the number of neutrophils by number of lymphocytes from peripheral blood sample. PFS was defined as the period calculated from the initiation of a single treatment to disease progression or death. The OS was defined as the period calculated from the initiation of the first-line EGFR-TKI treatment to date of death.

### Statistical analysis

Efficacy and prognosis were analysed for all patients who received afatinib, erlotinib, or gefitinib as first-line treatment and osimertinib, other EGFR-TKIs, or chemotherapeutic drugs as second-line therapy. The medians (ranges) of continuous variables with non-normal distributions are reported, whereas the frequencies (percentages) of categorical variables are reported. The Kruskal–Wallis test was used to compare continuous variables between different groups. The chi-square and Fisher’s exact tests were used to compare the efficacy between different subgroups. The median time to PFS or OS was calculated using the Kaplan–Meier method. Univariate and multivariate Cox regression analyses were used to evaluate the effects of clinical factors, including different first-line and second-line treatments, on PFS and OS of the patients. Hazard ratios (HR) with 95% confidence interval (95% CI) were calculated. All statistical analyses were performed using SPSS 25.0 and R 3.6.0 software. The level of statistical significance was set at *p* < 0.05.

## Results

A total of 242 patients were enrolled in this study. **Table 1** shows the demographic characteristics of the enrolled population. The median age was 66 years (range = 36 to 90 years). A higher proportion of the patients were female (51.5%), had never smoked (72.0%), and had a ECOG PS score of 0–1 (81.0%). Most patients had stage IV (88.4%) disease at diagnosis with < 3 metastatic sites (87.6%). In the case of metastases, 28.1% of the patients showed brain metastases and 12.8% liver metastases. An NLR of ≥5 was observed in 27.3% of all patients at disease progression on first-line treatment.

**Table 1:** Baseline characteristics of patients enrolled in this study.

Characteristic	Population (N=242)	Characteristic	Population (N=242)
**Age at diagnosis, median (range)**	66 (36, 90)		
**Gender**		**Brain metastasis at diagnosis**	68 (28.1%)
Male	117 (48.5%)	**Liver metastasis at diagnosis**	31 (12.8%)
Female	125 (51.5%)	**Metastatic sites**	
**Smoking status**		0	38 (15.7%)
Current smoker	34 (14.0%)	1	96 (39.7%)
Former smoker	34 (14.0%)	2	78 (32.2%)
Never smoked	174 (72.0%)	≥3	30 (12.4%)
**Staging at diagnosis**		**Neutrophil-to-lymphocyte ratio (NLR) at progression**	(n =188)
Stage III	28 (11.6%)	<5	133 (70.7%)
Stage IV	214 (88.4%)	≥5	55 (29.3%)
**ECOG Performance Status**		**First-line EGFR-TKI**	
0–1	196 (81.0%)	Gefitinib	70 (28.9%)
2–4	46 (19.0%)	Erlotinib	50 (20.7%)
**EGFR Mutation**		Afatinib	122 (50.4%)
Exon 19 deletion	109 (45.0%)	**Second-line regimen**	(n =206)
L858R mutation	120 (49.6%)	Osimertinib	18 (8.7%)
Uncommon mutations	13 (5.4%)	Other EGFR-TKIs	26 (12.6%)
**Re-biopsy after 1^st^-line progression**	70 (28.9%)	Chemotherapy	162 (78.6%)
**Re-biopsy after 2^nd^-line progression**	30 (11.5%)		

The median PFS and OS of the whole cohort were 19.1 (95% confidence interval [CI] = 17.7–20.5) months and 29.6 (95% CI = 27.1–32.1) months, respectively (**Supplementary Figure 1**). PFS and OS were better in patients that received afatinib as the first-line treatment (14.3 months and 34.1 months, respectively) than in those that received gefitinib (11.9 months and 25.8 months, respectively) or erlotinib (10.8 months and 26.7 months, respectively; **Supplementary Figure 2**).

**Figure 1:**
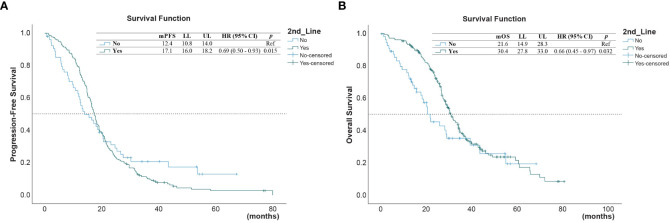
**(A)** Progression-free survival (PFS) and **(B)** overall survival (OS) in those who received second-line treatment after first-line treatment, and in those who did not*. (* reference).

Upon disease progress, 206 (85.1%) out of the 242 patients received second-line treatment, including 18 (8.7%) received osimertinib, 26 (12.6%) received EGFR-TKIs other than osimertinib, and 162 (78.6%) received chemotherapy. The survival outcomes of the second-line treatment are shown in **Table 2**. The median PFS and OS of the whole cohort were 5.03 (95% confidence interval [CI] = 4.47–5.60) months and 14.4 (95% CI = 12.80–16.0) months, respectively. A better PFS and OS were observed in patients who received second-line therapy than in those that did not (PFS 17.1 versus 12.4 months, *p* = 0.015; **Figure 1A**, and OS 30.4 versus 21.6 months, *p* = 0.032 for OS; **Figure 1B**).

**Table 2:** Analysis of lines of treatment and outcomes of patients on second-line treatment (n =206).

Subgroups	PFS						OS					
	N	No. of events	Median	0.95 LCL	0.95 UCL	*P-value*	N	No. of events	Median	0.95 LCL	0.95 UCL	*P-value*
**Whole cohort**	206	195	5.03	4.47	5.60		205	132	14.40	12.80	16.00	
Age												
<65 years	68	63	5.03	3.58	6.48	0.510	96	53	22.70	14.68	30.72	0.052
≥65 years	138	132	5.00	4.44	5.56		109	79	13.53	12.37	14.70	
Sex												
Female	96	88	5.13	3.73	6.54	0.047	96	53	15.87	11.94	19.79	0.098
Male	110	107	5.00	4.44	5.56		109	79	13.86	12.47	15.27	
ECOG												
0-1	171	161	5.07	4.36	5.78	0.013	170	111	14.23	12.51	15.96	0.957
≥2	35	34	4.33	2.88	5.79		35	21	15.03	9.04	21.03	
Smoking status												
Never smoker	139	128	5.13	4.58	5.69	0.009	138	88	14.40	12.61	16.19	0.407
Current	32	32	4.20	2.98	5.42		32	20	13.23	11.75	14.71	
Former	35	35	3.77	1.68	5.85		35	24	15.77	10.60	20.93	
Metastatic site												
<3	177	166	4.93	4.37	5.50	0.817	177	118	13.93	12.70	15.17	0.329
≥3	29	29	5.60	4.28	6.92		28	14	17.23	15.90	18.57	
Re-biopsy after first-line progression												
No	136	129	6.20	3.73	13.20	0.187	135	92	16.42	12.47	17.67	0.054
Yes	70	66	8.13	4.40	16.30		70	40	19.33	12.74	22.46	
Brain Metastasis												
No	150	142	5.10	4.31	5.89	0.038	150	96	14.40	12.38	16.42	0.527
Yes	56	53	4.20	2.94	5.46		55	36	14.10	10.20	18.00	
Liver Metastasis												
No	177	167	5.03	4.39	5.68	0.849	176	118	14.23	12.42	16.04	0.390
Yes	29	28	5.00	3.36	6.64		29	14	22.70	7.32	38.08	
Neutrophil-to-lymphocyte ratio (5.0)												
< 5.0	133	124	5.10	4.14	6.06	0.023	132	83	14.53	13.30	15.78	0.112
≥ 5.0	55	53	4.87	3.80	5.93		55	38	12.30	9.94	14.66	
Second-line treatment												
EGFR-TKI (others)	26	24	2.67	0.00	4.70	0.053	25	15	13.27	1.00	21.30	<0.001
Chemotherapy	162	156	5.00	4.70	6.60	0.123	159	108	14.27	27.90	35.30	0.021
EGFR-TKI (Osimertinib)	18	12	11.50	4.00	13.00		18	7	37.50	48.00	82.50	
Second-line treatment												
EGFR-TKI (others) + chemotherapy	188	180	4.87	4.30	5.50	0.002	184	123	14.27	13.30	32.50	0.001
EGFR-TKI (Osimertinib)	18	12	11.50	3.90	19.10		18	7	37.50	38.70	82.50	

*p < 0.05; PFS, progression-free survival; EGFR-TKI, epidermal growth factor receptor tyrosine kinase inhibitors; BSC, best supportive care.

**Figure 2:**
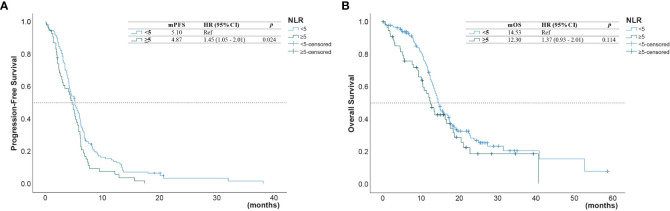
**(A)** Progression-free survival (PFS) and **(B)** overall survival (OS) of patients with a neutrophil-to-lymphocyte ratio (NLR) below 5.0 and those with an NLR above 5.0.

**Figure 3:**
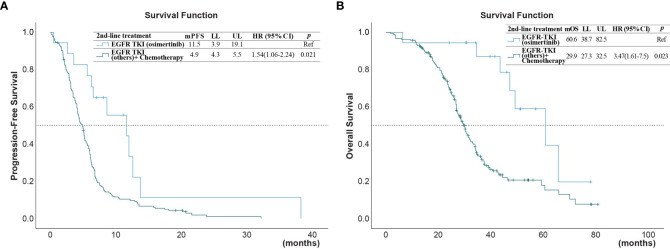
**(A)** Progression-free survival (PFS) and **(B)** overall survival (OS) of patients after second-line treatment with EGFR-TKI (osimertinib) versus treatments with chemotherapy or other EGFR-TKI (*reference group).

**Figure 4:**
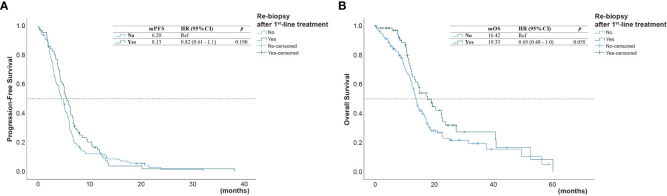
**(A)** Progression-free survival (PFS) and **(B)** overall survival (OS) of patients who underwent or did not undergo* re-biopsy after first-line progression (*reference group).

The univariate analysis (**Table 3**) showed that the PFS was shorter in male patients (1.3, 95% CI 1.0 - 1.78, p =0.049), patients with ECOG performance state ≥ 2 (HR 1.59, 95% CI 1.1 - 2.33, p =0.014), former smokers (1.79, 95% CI 1.23 - 2.63, p =0.003), patients with brain metastasis (HR 1.4, 95% CI 1.02-1.93, = 0.04), second-line with other EGFR-TKI or chemotherapy (HR 1.54, 95% CI 1.06-2.24, p =0.021). A NLR ≥5.0 was also associated with a shorter PFS (HR 1.45, 95% CI 1.05-2.01, p=0.024, **Figure 2A**), but not for OS (**Figure 2B**). Compared to second-line osimertinib, as shown in **Figure 3**, other EGFR-TKIs or chemotherapy was associated with a lower PFS (HR 1.54, 95% CI 1.06-2.24, p =0.021) and OS (HR 3.47, 95% CI 1.61-7.50, p =0.023). In the multivariate analysis, only second-line osimertinib was an independent predictor of better PFS (HR 2.74, 95% CI 1.32-5.69, p =0.007). OS was marginally longer in patients who underwent re-biopsy than in those that did not (19.33 versus 16.42 months, hazard ratio [HR] = 0.69, 95% CI = 0.48–1.0, p = 0.059; **Figure 4B**), but no significant difference for PFS (**Figure 4A**).

**Table 3:** Uni and multivariate Cox regression analysis for predictor of PFS on second-line treatment (n =206).

Variables	Univariate analysis		Multivariate analysis	
	HR (95% CI)	*P value*	Adjusted HR (95% CI)	*P value*
Age				
** <65 years old**	Ref		Ref	
** ≥65 years old**	1.11 (0.82 - 1.49)	0.512		
Sex				
** Female**	Ref		Ref	
** Male**	1.3 (1.0 - 1.78)	0.049*	3.37 (0.68 - 16.61)	0.136
ECOG PS				
** 0-1**	Ref		Ref	
** ≥2**	1.59 (1.1 - 2.33)	0.014*	0.46 (0.09 - 2.36)	0.352
Smoking status				
** Never smoker**	Ref		Ref	
** Current smoker**	1.19 (081 - 1.77)	0.369	—	
** Former smoker**	1.79 (1.23 - 2.63)	0.003*	2.8 (.36 - 20.97)	0.329
Metastatic sites				
** <3**	Ref		Ref	
** ≥3**	0.97 (0.65 - 1.45)	0.87	—	
Re-biopsy after 1st-line progression				
** No**	Ref		Ref	
** Yes**	0.82 (0.61 - 1.1)	0.190	—	
Brain Metastasis				
** No**	Ref		Ref	
** Yes**	1.4 (1.02 -1.93)	0.04*	1.52 (0.42 - 5.58)	0.529
Liver Metastasis				
** No**	Ref		Ref	
** Yes**	1.04 (0.69 - 1.6)	0.850		
Second-line treatment				
** EGFR-TKI (osimertinib)**	Ref		Ref	
** EGFR-TKI (others)**	2.64 (1.28 - 5.42)	0.008	2.45 (0.99 - 6.09)	0.053
** Chemotherapy**	2.68 (1.45 - 4.97)	0.002	1.86 (0.85 - 4.07)	0.123
Second-line treatment				
** EGFR-TKI (osimertinib)**	Ref		Ref	
** EGFR-TKI (others) + chemotherapy**	1.54 (1.06 - 2.24)	0.021	2.74 (1.32 - 5.69)	0.007*
NLR				
** <5**	Ref		Ref	
** ≥5**	1.45 (1.05 - 2.01)	0.024*	2.95 (0.61 - 14.37)	0.180

*p < 0.05; PFS, progression-free survival; EGFR-TKI, epidermal growth factor receptor tyrosine kinase inhibitors.

**Table 4** shows the univariate and multivariate analyses for the predictors of OS. Patients with NLR ≥5.0 had shorter OS than patients with NLR <5.0 (12.3 versus 14.5 months, HR =1.66, 95% CI 1.14-2.42, p = 0.008; **Figure 2A**). No significant associations were found between survival outcomes (PFS and OS) with ECOG PS, no metastasis sites, mutation type, and time of diagnosis.

**Table 4:** Uni and multivariate Cox regression analysis for predictor of OS on second-line treatment (n =206).

Variables	Univariate analysis		Multivariate analysis	
	HR (95% CI)	*P value*	Adjusted HR (95% CI)	*P value*
Age				
** <65 years old**	Ref		Ref	
** ≥65 years old**	1.46 (0.99 - 2.14)	0.054	—	
Sex				
** Female**	Ref		Ref	
** Male**	0.86 (0.72 - 1.03)	0.10	—	
ECOG PS				
** 0-1**	Ref		Ref	
** ≥2**	1.01 (0.63 - 1.63)	0.957	—	
Smoking status				
** Never smoker**	Ref		Ref	
** Current smoker**	0.86 (0.52 - 1.43)	0.586	—	
** Former smoker**	1.29 (0.82 - 2.02)	0.279	—	
Metastatic sites				
** <3**	Ref		Ref	
** ≥3**	0.76 (0.44 - 1.32)	0.331	—	
Re-biopsy after 1st-line progression				
** No**	Ref		Ref	
** Yes**	0.69 (0.48 - 1.0)	0.059	—	
Brain Metastasis				
** No**	Ref		Ref	
** Yes**	1.13 (0.77 - 1.67)	0.528	—	
Liver Metastasis				
** No**	Ref		Ref	
** Yes**	0.78 (0.45 - 1.37)	0.394	—	
Second-line treatment				
** EGFR-TKI (osimertinib)**	Ref		Ref	
** EGFR-TKI (others)**	3.9 (1.59 - 9.6)	0.003	—	
** Chemotherapy**	3.47 (1.61 - 7.5)	0.002	—	
Second-line treatment				
** EGFR-TKI (osimertinib)**	Ref		Ref	
** EGFR-TKI (others) + chemotherapy**	3.47 (1.61 – 7.5)	0.023	—	
NLR				
** <5**	Ref		Ref	
** ≥5**	1.37 (0.93 - 2.01)	0.114	—	

*p < 0.05; PFS, progression-free survival; EGFR-TKI, epidermal growth factor receptor tyrosine kinase inhibitors.

## Discussion

In this multicenter retrospective study, we investigated the factors associated with survival outcomes of second-line treatments for *EGFR*-mutant NSCLC in patients with disease progression on first- or second-generation EGFR-TKI in Taiwan. Our study showed that PFS was shorter in male gender, ECOG status ≥2, former smoking, brain metastasis, second-line chemotherapy or EGFR-TKIs other than osimertinib, and NLR ≥5.0. The multivariate analysis indicated osimertinib as the second-line treatment was an independent predictor of PFS. These results suggest that the benefits of osimertinib necessitates that aggressive re-biopsy after progression on first- or second-generation EGFR-TKI treatment is merited for appropriate second-line treatments to provide better outcomes for these patients.

EGFR-TKI therapy is the standard treatment for patients with advanced *EGFR*-mutated NSCLC; it has a higher response rate and provides better symptom control and quality of life improvements compared to conventional chemotherapy or immunotherapy.(15, 16) However, disease progression with acquired resistance is inevitable and tissue re-biopsy or liquid biopsy is recommended for the guide of second-line treatment. Nevertheless, the re-biopsy rate after disease progression on first-line EGFR-TKI is variable in real-world studies, ranging from 60% to 90%.(17–20) The low re-biopsy rate (28.9%) in this study is likely due to osimertinib not been reimbursed by the National Institutes of Health (NIH) of Taiwan until early 2020. This might have led to fewer patients consenting to re-biopsy and reduced clinicians’ willingness to perform it, since most patients may not be able to afford osimertinib as a subsequent treatment even when the outcome is positive for the t790M mutation. However, PFS and OS were significantly longer in those patients receiving osimertinib as a second-line treatment than in those who received other treatments or did not receive any subsequent treatment. This result is comparable to or even better than the results of previous retrospective real-world studies, which showed a PFS of 9.4 to 10.1 months and OS of 24 to 47.3 months for osimertinib as a second-line or subsequent treatment.(21–23) The better PFS and OS in this study may be due to the relatively small number of patients and the focus on only those treated with osimertinib as the second-line therapy.

For NSCLC patients without the T790M mutation, platinum-based chemotherapy is the recommended second-line therapy after progression on first- and second-generation EGFR-TKIs.(24) Nevertheless, a randomised phase 2 trial of 96 patients in Korea concluded that the outcomes of pemetrexed therapy for NSCLC patients with disease progression after first-line EGFR-TKI were not improved by adding cisplatin.(25) Thus, which chemotherapeutic regimen was optimal as the treatment standard was unclear. Our study found that different chemotherapeutic regimens resulted in similar PFS and OS. A previous meta-analysis, which included one randomised controlled trial and three retrospective studies, showed that second-line treatment with pemetrexed chemotherapeutic regimens provided significantly longer PFS and OS than non-pemetrexed chemotherapeutic regimens.(26) However, this meta-analysis was greatly limited by the small sample size; therefore, its results should be interpreted cautiously. Additional well-designed prospective studies are warranted to resolve this controversial issue.

There is increasing evidence that NLR, a surrogate of inflammatory and immunologic indicators, is an independent predictor of poor prognosis in cancer patients, including those with NSCLC.(27, 28) A retrospective study of 190 metastatic NSCLC patients receiving EGFR-TKIs indicated that a higher NLR was associated with poor prognosis.(27) Recently, a pooled analysis of two phase III NSCLC clinical trial datasets also indicated that a higher baseline NLR (≥3.8) was associated with worse PFS (HR = 1.37, *p* = 0.0004) and OS (HR = 1.65, *p* < 0.0001).(28) Our results are in agreement with these previous findings. NLR is an easily accessible and effective prognostic biomarker for NSCLC patients. However, the specific mechanism of its prognostic value is not clear. Further well-designed studies are required to modify the prognostic role of NLR in lung cancer patients.

One of the limitations of this study is that the retrospective nature of real-world, population-based settings tend to generate selection bias when the study population is examined using different patient characteristics. For example, patients with good performance status and an easily accessible tumor site are more likely to receive re-biopsy or second-line treatment, whereas those with rapid disease progression or an unavailable tumor site (such as progression with brain metastases) are less likely to undergo re-biopsy or subsequent treatment. In addition, liquid biopsy is not covered by Taiwan’s NIH; therefore, only some patients being able to afford the test and further treatment could affect survival outcomes. Furthermore, the sample size in this study was relatively small, which may also introduce bias and limit the possibility of highlighting general implications. Finally, we did not include patients with acquired T790M mutation after re-biopsy as an endpoint, so the information on the treatment for patients with acquired T790M mutation after re-biopsy and the reasons for patients not receiving re-biopsy were not available in the present study. However, it is reasonable according to the statement from American Society of Clinical Oncology (29), which indicated that the key elements of framework are the clinical benefits (e.g., hazard ratio for death, overall survival, and progression-free survival).

## Conclusion

The results of this study indicated PFS was shorter in male gender, ECOG status ≥2, former smoking, brain metastasis, second-line with EGFR-TKI other than osimertinib or chemotherapy, and NLR ≥5.0. Osimertinib as the second-line treatment was an independent predictor of PFS. These results suggest that the benefits of osimertinib necessitates that aggressive re-biopsy after progression on first- or second-generation EGFR-TKI treatment is merited for appropriate second-line treatments to provide better outcomes for these patients.

## Data availability statement

The original contributions presented in the study are included in the article/**Supplementary Material**. Further inquiries can be directed to the corresponding author.

## Ethics statement

The studies involving human participants were reviewed and approved by E-Da Hospital EMRP-110-080, National Taiwan University Hospital NTUH-201611059RINB, Far Eastern Memorial Hospital FEMH-111075-E, and Yang-Ming Chiao Tung University Hospital YMUH-2021A022. Written informed consent for participation was not required for this study in accordance with the national legislation and the institutional requirements.

## Author contributions

Conception and design: Che-YC, Chu-YC, S-CC, Chi-YC, and Y-FW; Acquisition of data: Che-YC, Chu-YC, Chi-YC, Y-CL, and C-FC; Analysis and interpretation of data: Che-YC, Chu-YC, S-CC, Chi-YC, Y-CL, C-FC, and Y-FW; Drafting the article: Che-YC, Chu-YC, S-CC, and Y-FW; Revising the article critically for important intellectual content: Che-YC, Chu-YC, S-CC, Chi-YC, Y-CL, C-FC, and Y-FW.

## References

[1] Sung H Ferlay J Siegel RL Laversanne M Soerjomataram I Jemal A. Global cancer statistics 2020: GLOBOCAN estimates of incidence and mortality worldwide for 36 cancers in 185 countries. CA Cancer J Clin (2021) 30(Supplement_9). doi: 10.3322/caac.21660 33538338

[2] Riessk J. Shifting paradigms in non-small cell lung cancer: an evolving therapeutic landscape. Am J Manag Care (2013) 19:s390–397. doi: 10.6004/jnccn.2021.0013 24494720

[3] Shi Y Li J Zhang S Wang M Yang S Li N. Molecular epidemiology of EGFR mutations in Asian patients with advanced non-Small-Cell lung cancer of adenocarcinoma histology - mainland China subset analysis of the PIONEER study. PloS One (2015) 10:e0143515. doi: 10.1371/journal.pone.0143515 26599344 PMC4657882

[4] Mok TS Wu YL Thongprasert S Yang CH Chu DT Saijo N. Gefitinib or carboplatin-paclitaxel in pulmonary adenocarcinoma. N Engl J Med (2009) 361:947–57. doi: 10.1056/NEJMoa0810699 19692680

[5] Rosell R Carcereny E Gervais R Vergnenegre A Massuti B Felip E. Erlotinib versus standard chemotherapy as first-line treatment for European patients with advanced EGFR mutation-positive non-small-cell lung cancer (EURTAC): a multicentre, open-label, randomised phase 3 trial. Lancet Oncol (2012) 13:239–46. doi: 10.1016/S1470-2045(11)70393-X 22285168

[6] Yang JC Wu YL Schuler M Sebastian M Popat S Yamamoto N. Afatinib versus cisplatin-based chemotherapy for EGFR mutation-positive lung adenocarcinoma (LUX-lung 3 and LUX-lung 6): analysis of overall survival data from two randomised, phase 3 trials. Lancet Oncol (2015) 16:141–51. doi: 10.1016/S1470-2045(14)71173-8 25589191

[7] Ettinger DS Wood DE Aisner DL Akerley W Bauman JR Bharat A. NCCN guidelines insights: non-small cell lung cancer, version 2.2021. J Natl Compr Canc Netw (2021) 19:254–66. doi: 10.6004/jnccn.2021.0013 33668021

[8] Ramalingam SS Vansteenkiste J Planchard D Cho BC Gray JE Ohe Y. Overall survival with osimertinib in untreated, EGFR-mutated advanced NSCLC. N Engl J Med (2020) 382:41–50. doi: 10.1056/NEJMoa1913662 31751012

[9] Tsukita Y Inoue A. First-line therapy in non-small cell lung cancer patients with EGFR activating mutations: a consideration of the clinical position of osimertinib based on the subset of Japanese patients in the FLAURA study. Jpn J Clin Oncol (2022) 13:1758835921996509. doi: 10.1093/jjco/hyac012 35446957

[10] Papadimitrakopoulou VA Mok TS Han JY Ahn MJ Delmonte A Ramalingam SS. Osimertinib versus platinum-pemetrexed for patients with EGFR T790M advanced NSCLC and progression on a prior EGFR-tyrosine kinase inhibitor: AURA3 overall survival analysis. Ann Oncol (2020) 31:1536–44. doi: 10.1016/j.annonc.2020.08.2100 32861806

[11] Wu YL Planchard D Lu S Sun H Yamamoto N Kim DW. Pan-Asian adapted clinical practice guidelines for the management of patients with metastatic non-small-cell lung cancer: a CSCO-ESMO initiative endorsed by JSMO, KSMO, MOS, SSO and TOS. Ann Oncol (2019) 30:171–210. doi: 10.1093/annonc/mdy554 30596843

[12] Seto T Nogami N Yamamoto N Atagi S Tashiro N Yoshimura Y. Real-world EGFR T790M testing in advanced non-Small-Cell lung cancer: a prospective observational study in Japan. Oncol Ther (2018) 6:203–15. doi: 10.1007/s40487-018-0064-8 PMC7359964 32700028

[13] Magios N Bozorgmehr F Volckmar AL Kazdal D Kirchner M Herth FJ. Real-world implementation of sequential targeted therapies for EGFR-mutated lung cancer. Ther Adv Med Oncol (2021) 13:1758835921996509. doi: 10.1177/1758835921996509 34408792 PMC8366107

[14] Mountzios G Koumarianou A Bokas A Mavroudis D Samantas E Fergadis EG. A real-world, observational, prospective study to assess the molecular epidemiology of epidermal growth factor receptor (EGFR) mutations upon progression on or after first-line therapy with a first- or second-generation EGFR tyrosine kinase inhibitor in EGFR mutation-positive locally advanced or metastatic non-small cell lung cancer: the 'LUNGFUL' study. Cancers (Basel) (2021) 13(1):230. doi: 10.3390/cancers13133172 PMC8268841 34202063

[15] Geater SL Xu CR Zhou C Hu CP Feng J Lu S. Symptom and quality of life improvement in LUX-lung 6: an open-label phase III study of afatinib versus Cisplatin/Gemcitabine in Asian patients with EGFR mutation-positive advanced non-small-cell lung cancer. J Thorac Oncol (2015) 10:883–9. doi: 10.1097/JTO.0000000000000517 25933111

[16] Shi C Wang Y Xue J Zhou X. Immunotherapy for EGFR-mutant advanced non-small-cell lung cancer: current status, possible mechanisms and application prospects. Front Immunol (2022) 13:940288. doi: 10.3389/fimmu.2022.940288 35935943 PMC9353115

[17] Ahn H Kim Y Kim EY Kim KW Sung KH Cho EK. Feasibility of rebiopsy and sequential treatment of EGFR tyrosine kinase inhibitors in real world patients with EGFR mutant non-small cell lung cancer. Ann Oncol (2019) 30:ix166. doi: 10.1093/annonc/mdz437.018

[18] Hong MH Kim HR Ahn BC Heo SJ Kim JH Cho BC. Real-world analysis of the efficacy of rebiopsy and EGFR mutation test of tissue and plasma samples in drug-resistant non-small cell lung cancer. Yonsei Med J (2019) 60:525–34. doi: 10.3349/ymj.2019.60.6.525 PMC6536394 31124335

[19] Shah R Girard N Nagar SP Griesinger F Roeper J Davis KL. European And US real-world treatment patterns in patients with epidermal growth factor receptor mutation-positive non-small cell lung cancer: a retrospective medical record review. Drugs Real World Outcomes (2021) 8:537–45. doi: 10.1007/s40801-021-00261-8 PMC8605952 34533784

[20] Koyama K Miura S Watanabe S Shoji S Koshio J Hayashi Y. Observational study of rebiopsy in EGFR-TKI-resistant patients with EGFR mutation-positive advanced NSCLC. Sci Rep (2022) 12:6367. doi: 10.1038/s41598-022-10288-8 35430596 PMC9013397

[21] Stratmann JA Michels S Hornetz S Christoph DC Sackmann S Spengler W. Efficacy and safety analysis of the German expanded access program of osimertinib in patients with advanced, T790M-positive non-small cell lung cancer. J Cancer Res Clin Oncol (2018) 144:2457–63. doi: 10.1007/s00432-018-2754-x PMC11813429 30244389

[22] Mehlman C Cadranel J Rousseau-Bussac G Lacave R Pujals A Girard N. Resistance mechanisms to osimertinib in EGFR-mutated advanced non-small-cell lung cancer: a multicentric retrospective French study. Lung Cancer (2019) 137:149–56. doi: 10.1016/j.lungcan.2019.09.019 31600593

[23] Provencio M Terrasa J Garrido P Campelo RG Aparisi F Diz P. Osimertinib in advanced EGFR-T790M mutation-positive non-small cell lung cancer patients treated within the special use medication program in Spain: OSIREX-Spanish lung cancer group. BMC Cancer (2021) 21:230. doi: 10.1186/s12885-021-07922-5 33676426 PMC7937205

[24] Liao BC Griesing S Yang JC. Second-line treatment of EGFR T790M-negative non-small cell lung cancer patients. Ther Adv Med Oncol (2019) 11:1758835919890286. doi: 10.1177/1758835919890286 31803256 PMC6878608

[25] Yoo KH Lee SJ Cho J Lee KH Park KU Kim KH. A randomized, open-label, phase II study comparing pemetrexed plus cisplatin followed by maintenance pemetrexed versus pemetrexed alone in patients with epidermal growth factor receptor (EGFR)-mutant non-small cell lung cancer after failure of first-line EGFR tyrosine kinase inhibitor: KCSG-LU12-13. Cancer Res Treat (2019) 51:718–26. doi: 10.4143/crt.2018.324 PMC6473296 30177585

[26] Li Z Guo H Lu Y Hu J Luo H Gu W. Chemotherapy with or without pemetrexed as second-line regimens for advanced non-small-cell lung cancer patients who have progressed after first-line EGFR TKIs: a systematic review and meta-analysis. Onco Targets Ther (2018) 11:3697–703. doi: 10.2147/OTT.S160147 PMC6027845 29983578

[27] Xie X Li X Tang W Chen J Xie P Li M. Prognostic value of the neutrophil-to-lymphocyte ratio and primary tumor location in epidermal growth factor receptor-mutated metastatic non-small cell lung cancer. J Cancer Res Ther (2021) 17:1618–25. doi: 10.4103/jcrt.jcrt_1442_21 35381730

[28] Huang L Jiang S Shi Y. Prognostic significance of baseline neutrophil-lymphocyte ratio in patients with non-small-cell lung cancer: a pooled analysis of open phase III clinical trial data. Future Oncol (2022) 18:1679–89. doi: 10.2217/fon-2021-1304 35132871

[29] Schnipper LE Davidson NE Wollins DS Blayney DW Dicker AP Ganz PA. Updating the American society of clinical oncology value framework: revisions and reflections in response to comments received. J Clin Oncol (2016) 34:2925–34. doi: 10.1200/JCO.2016.68.2518 27247218

