# Supplementary figures and images for: Factors associated with outcomes of second-line treatment for EGFR-mutant non-small-cell lung cancer patients after progression on first- or second-generation EGFR-tyrosine kinase inhibitor treatment
Source: Front Oncol. 2023 Jun 20;13:1104098. doi: 10.3389/fonc.2023.1104098 (PMC10318893; doi:10.3389/fonc.2023.1104098)

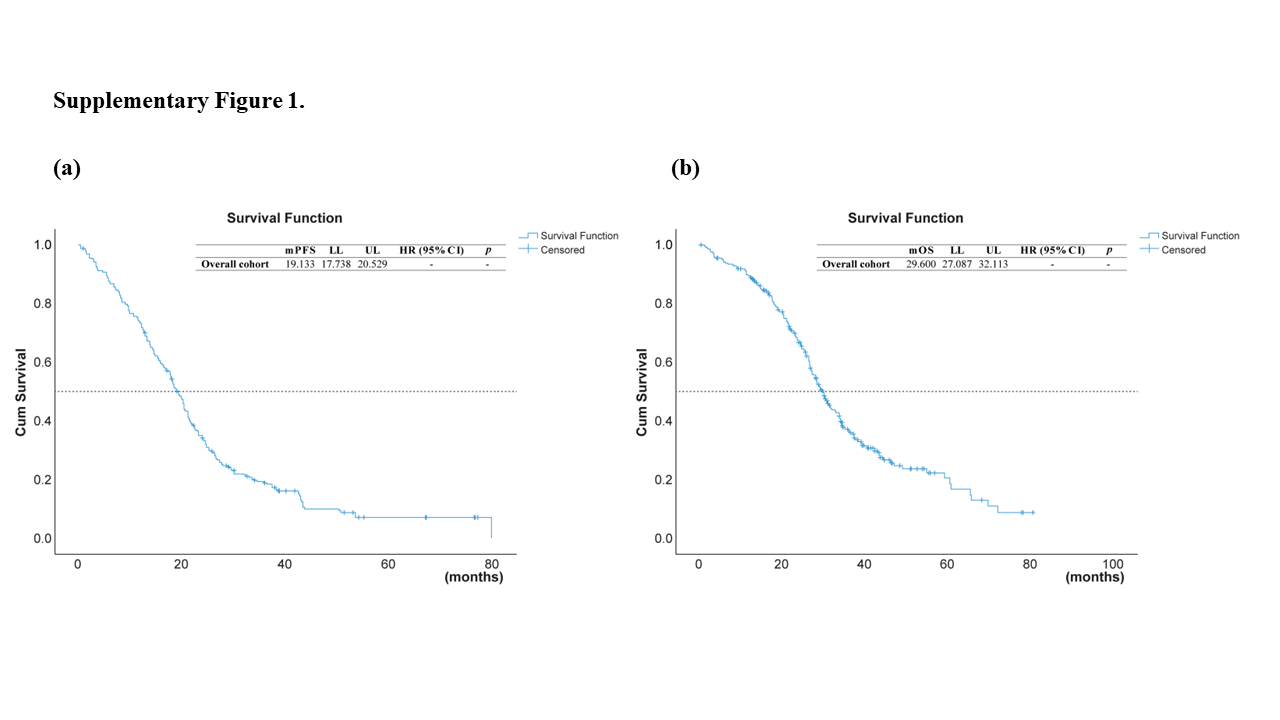

Supplement: Supplementary file 1 [file Image_1.tif]

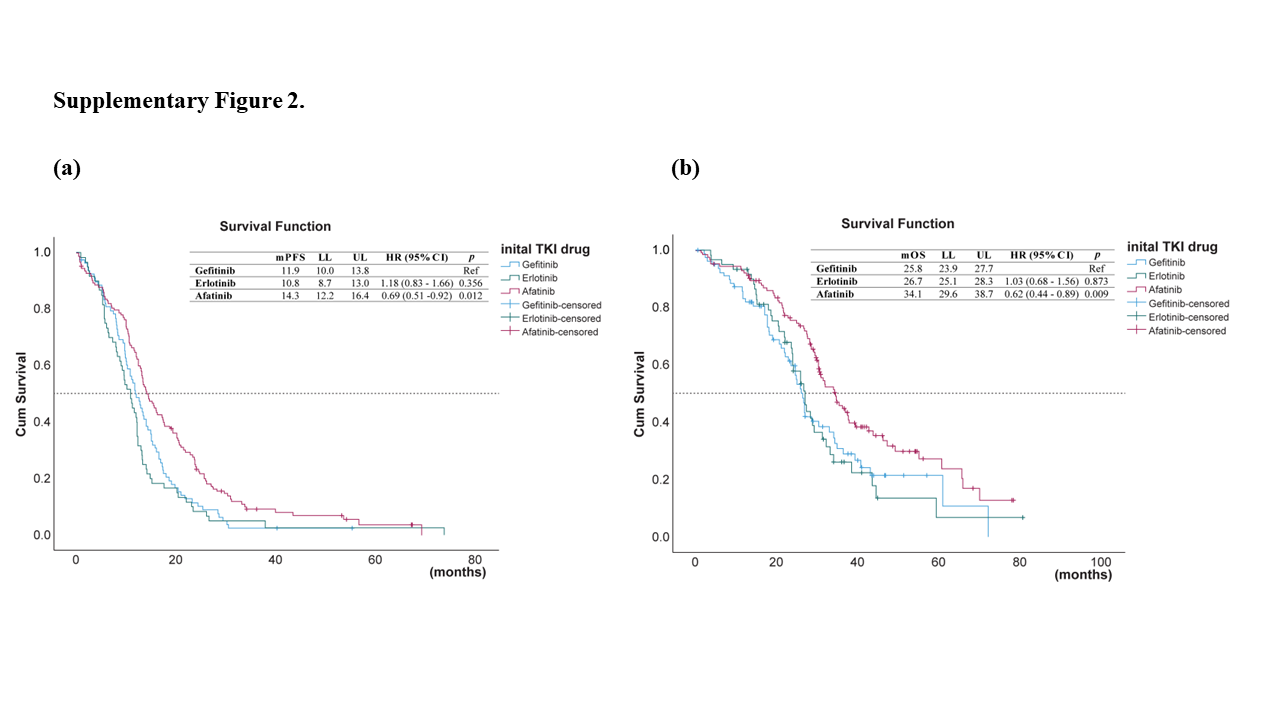

Supplement: Supplementary file 2 [file Image_2.tif]
